# Integration of transbronchial cryobiopsy into multidisciplinary board decision: a single center analysis of one hundred consecutive patients with interstitial lung disease

**DOI:** 10.1186/s12931-021-01821-w

**Published:** 2021-08-14

**Authors:** Katrin E. Hostettler, Michael Tamm, Lukas Bubendorf, Peter Grendelmeier, Kathleen Jahn, Daiana Stolz, Jens Bremerich, Spasenija Savic Prince

**Affiliations:** 1 Clinics of Respiratory Medicine, University Hospital Basel, Petersgraben 4, 4031 Basel, Switzerland; 2 Pathology, Institute of Medical Genetics and Pathology, University Hospital Basel, 4054 Basel, Switzerland; 3 Department of Radiology, University Hospital Basel, 4031 Basel, Switzerland

**Keywords:** Cryobiopsy, Interstitial lung disease, Multidisciplinary team discussion

## Abstract

**Background:**

Transbronchial cryobiopsy in the evaluation of patients with interstitial lung diseases (ILD) is expected to reduce the need for surgical lung biopsy (SLB).

**Objective:**

To evaluate the diagnostic value of cryobiopsy in combination with bronchoalveolar lavage (BAL), radiologic and clinical data in patients with ILD.

**Methods:**

Between 08/15 and 01/20 patients with ILD underwent cryobiopsy if they: did *not* have (i) an usual interstitial pneumonia (UIP)-pattern on CT, (ii) predominant ground-glass opacities suggesting alveolitis, (iii) findings suggestive of sarcoidosis on CT, or if they *had* (i) a CT showing UIP-pattern, but had findings suggesting alternative diagnosis than idiopathic pulmonary fibrosis (IPF), or (ii) had previous non-diagnostic conventional transbronchial forceps biopsy. Histological findings were integrated into the multidisciplinary team discussion (MDTD) and a diagnostic consensus was sought.

**Results:**

One hundred patients underwent cryobiopsy. In 88/100 patients, cryobiopsy was representative with diagnostic findings in 45/88 and non-specific histological findings in 43/88 patients. In 25/43 with non-specific findings, a consensus diagnosis was reached after MDTD integrating BAL, radiologic and clinical data; eight of the remaining 18 patients with non-specific findings were referred to SLB. In 12/100 patients cryobiopsy was not representative and three of these patients were also referred to SLB. In 7/11 patients (64%) SLB was diagnostic. Complications of cryobiopsy included pneumothorax (14%) and locally controlled bleeding (24%).

**Conclusions:**

The diagnostic yield of cryobiopsy was 70%:45% of cryobiopsies were diagnostic based on histology alone and an additional 25% provided non-specific, but valuable findings allowing a consensus diagnosis after MDTD. Our data demonstrate that the diagnostic value of cryobiopsy is high if combined with BAL, radiologic and clinical data.

## To the Editor

Transbronchial cryobiopsy has emerged as a new diagnostic tool in the evaluation of patients with interstitial lung diseases (ILD) and is expected to reduce the need for surgical lung biopsy (SLB). A high level (69.2%) of histopathological agreement between cryobiopsy and SLB has been shown in a recent prospective study [1]. Another recent prospective multicenter study demonstrated that cryobiopsy—added to clinico-radiological and broncho-alveolar lavage (BAL) data—improved the level of diagnostic confidence for patients with suspected ILD [2]. In our hospital, cryobiopsy was integrated in the diagnostic work-up of patients with suspected ILD in August 2015. Here, we report the diagnostic performance of cryobiopsy in combination with BAL, radiological and clinical data in 100 consecutive patients in a real-world multidisciplinary team discussion (MDTD)-setting.

Between August 2015 and January 2020, patients with suspected ILD underwent cryobiopsy if they: did *not* have (i) a usual interstitial pneumonia (UIP)-pattern on CT, (ii) predominant ground-glass opacities suggesting alveolitis, (iii) findings suggestive of sarcoidosis on CT, or if they *had* (i) a CT showing a UIP-pattern, but additional findings suggesting a diagnosis other than idiopathic pulmonary fibrosis (IPF), or (ii) had a previous non-diagnostic conventional transbronchial forceps biopsy.

CT scans for all patients were classified into one of the following radiological categories: typical UIP pattern, probable UIP pattern, indeterminate pattern, and alternative diagnosis pattern [3]. Cryobiopsies were performed by flexible bronchoscopy, under fluoroscopic guidance in sedated, spontaneously breathing patients. To reduce bleeding risk, anticoagulants and adenosine-diphosphate receptor antagonists were discontinued prior to the intervention. Treatment with acetylsalicylic acid was not interrupted. A flexible endotracheal tube was positioned via the bronchoscope. In order to reduce post-interventional bleeding, an endobronchial balloon blocker was placed in the bronchus leading to the biopsied area and inflated after each cryobiopsy. Prophylactic endobronchial application of 1 mg terlipressin in the respective bronchus was performed and repeated in case of moderate bleeding. A minimum of two biopsies from different segments of the same lobe were taken. Probe sizes of 1.9 or 2.4 mm were used and activated for 3–4 s. The cryoprobe, attached lung tissue and the bronchoscope were removed together, whilst the balloon catheter stayed in place. To exclude pneumothorax, fluoroscopic control was done at the end of the intervention, and a chest X-ray was performed within 24 h after the intervention. All patients stayed hospitalized for a minimum of one night after cryobiopsy. Cryobiopsies were histologically evaluated by experienced lung pathologists specialized in ILD (LB or SSP). At time of initial cryobiopsy evaluation at the microscope, the pathologists had additional information from the BAL and the brief clinical information from the ordering sheet. Cryobiopsy findings were integrated with BAL-, CT- and clinical findings during the MDTD and a diagnostic consensus was sought. For this retrospective analysis, the initial histopathological findings/diagnoses from the written reports were collected and categorized into the following groups: (i) not representative findings (no lung parenchyma), (ii) sampling error (normal lung parenchyma, not representative of the CT findings), (iii) non-specific findings, not allowing for a narrow differential diagnosis, (iv) findings highly compatible with a specific disease in the appropriate clinical setting, e.g. compact granuloma for sarcoidosis or loose granuloma with bronchiolocentric inflammation for hypersensitivity pneumonitis, and (v) specific/diagnostic findings, as in case of Langerhans cell histiocytosis, lipoid pneumonia, cancer, or pulmonary alveolar proteinosis. Initial histopathological diagnosis based on cryobiopsy and the subsequent consensus-diagnosis during MDTD were assessed; SLB results (if performed) were compared with cryobiopsy findings and MDTD reports.

**Fig. 1:**
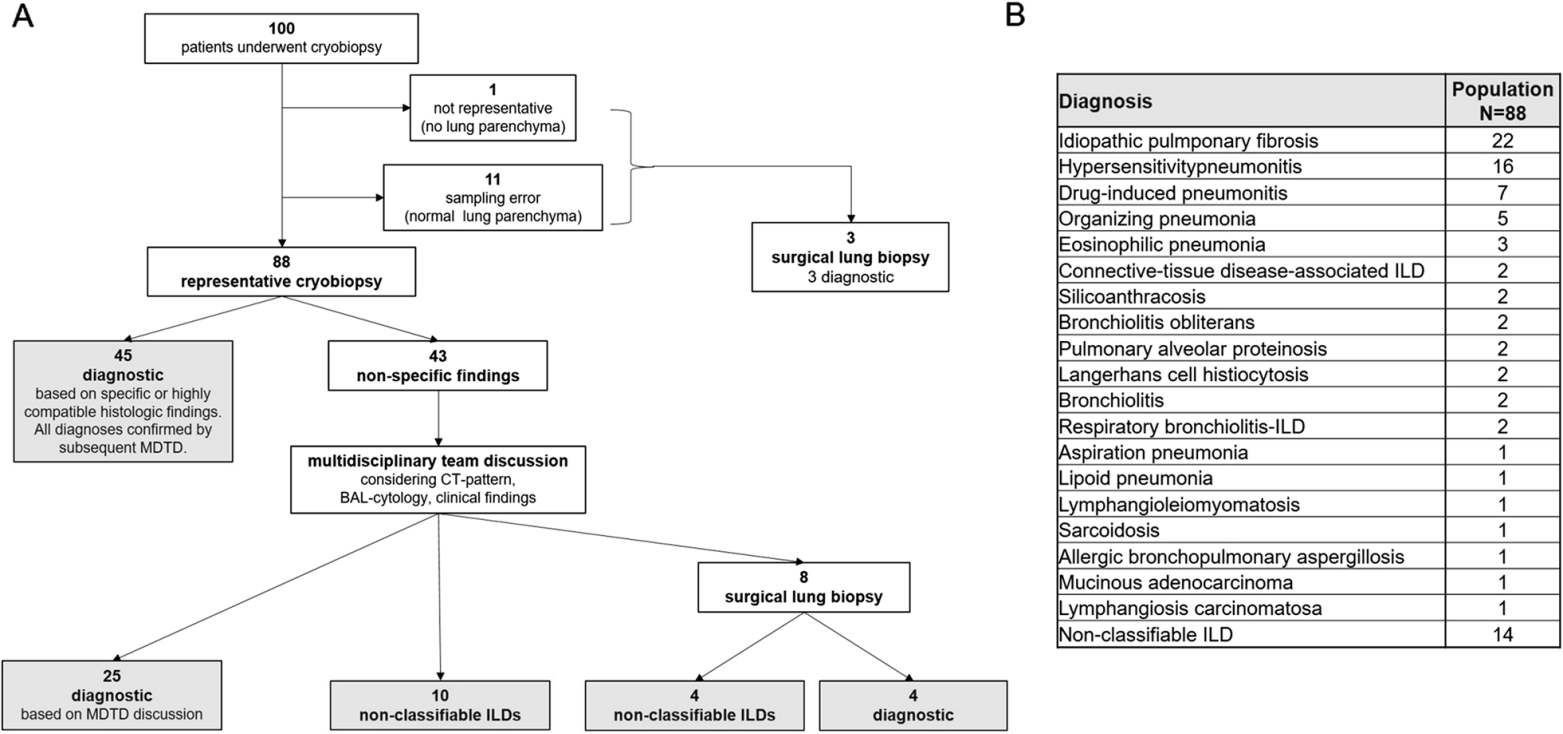
**A** One hundred patients undergoing transbronchial cryobiopsy. Definition of abbreviations: *BAL* bronchoalveolar lavage; *CT* computed tomography; ILD interstitial lung disease; *MDTD* multidisciplinary team discussion. **B** Final diagnosis of all patients with representative cryobiopsy (n = 88). Definition of abbreviations: *ILD* interstitial lung disease

A total of 100 consecutive patients underwent cryobiopsy; the mean age was 65 years (± 13), 63 were of male sex (63%), and the mean percentage predicted values for forced vital capacity and diffusing capacity of the lung for carbon were 84% (± 18) and 61% (± 18), respectively. In 88/100 patients (88%), cryobiopsy was representative, i.e. it successfully captured diseased lung parenchyma (Fig. 1A); in 12/100 patients (12%) cryobiopsy was not representative due to sampling error (normal lung parenchyma; n = 11) or due to non-representative biopsies (only bronchial tissue; n = 1). Among patients with representative cryobiopsy, initial evaluation by the pathologists revealed specific/diagnostic findings or findings highly compatible with a specific disease in 45/88 patients (51%). All these histopathological diagnoses were confirmed by the subsequent consensus MDTD-diagnoses and therefor allowed for a specific and confident diagnosis. In 43/88 (49%) patients with representative cryobiopsy, the histological findings were non-specific (Fig. 1A). In 25/43 patients (51.1%) with non-specific findings, a consensus diagnosis was reached after MDTD integrating cryobiopsy, BAL, radiologic and clinical findings. Eight of the remaining 18 patients with non-specific cryobiopsy findings were referred to SLB, whereas in 10 patients—due to age and/or comorbidities—no further diagnostic work-up was performed, and diagnosis of non-classifiable ILD was agreed on. In three of 12 patients with non-representative cryobiopsy, SLB was performed, whereas in nine patients SLB was withhold due to relevant comorbidities and/or old age. Thus, a total of 11/100 patients (11%) received SLB. In 7/11 patients (64%), SLB was diagnostic. In patients with non-diagnostic cryobiopsy who underwent SLB (n = 8), the final diagnoses were IPF (n = 2), hypersensitivity pneumonitis (HP) (n = 1), pulmonary alveolar proteinosis (n = 1), and in four patients SLB equally showed a non-classifiable ILD. Postinterventional pneumothorax occurred in 14 patients (14%), requiring chest tube drainage in ten of them. Locally controlled bleeding after cryobiopsy was observed in 24% of patients; there was no case of fatal bleeding.

Among patients with a representative cryobiopsy (n = 88), CT scan showed a typical UIP pattern in 13 (15%), a probable UIP pattern in 7 (8%), and a pattern suggestive of an *alternative diagnosis* in 68 patients (77%). As mentioned above, in all patients with a radiological UIP pattern, there were additional findings suggestive of a non-IPF diagnosis (e.g. young age, environmental exposure to allergens suggesting chronic HP).

Among 68 patients with an alternative diagnosis pattern on CT scan, cryobiopsy lead to a diagnosis in 52 patients (76%), including 10 patients (15%) with a final diagnosis of IPF. In those 13 patients with a typical UIP pattern on CT scan, cryobiopsy allowed a confident diagnosis in 12 patients (5 IPF, 4 HP, 1 drug-induced pneumonitis, 1 eosinophilic pneumonia, 1 connective-tissue disease-associated ILD), whereas one patient was diagnosed as having non-classifiable ILD. All patients undergoing SLB (n = 11) had a chest CT scan showing an alternative diagnosis pattern.

In conclusion, in this analysis of 100 consecutive patients the diagnostic yield of cryobiopsy in combination with BAL, radiological and clinical findings was 70%:45% of cryobiopsies were diagnostic based on histology alone and an additional 25% provided non-specific, but valuable histologic findings allowing to narrow down the differential diagnosis and to reach a consensus diagnosis after MDTD.

The combination of clinical, radiological and pathological findings is the principal task of the MDTD [4]. If clinical, serological, radiological, and BAL findings do not provide sufficient information for establishing a confident diagnosis, tissue biopsy is recommended [3]; SLB is still considered the gold standard [3]. SLB provides a high diagnostic yield around 95% [3], but due to the non-negligible procedure-related mortality [5], the individual benefit-risk-ratio has to be weighed carefully.

Our data are in line with a previous retrospective analysis of 74 patients with ILD undergoing cryobiopsy reporting a diagnostic yield of 51%, which further increased to 79.7% after additional MDTD [6]. Importantly, a recently published prospective multicenter study by Hetzel et al. demonstrated that cryobiopsy—added to clinico-radiological and broncho-alveolar lavage (BAL) data—led to an increase in diagnostic confidence from 60.2 to 81.2%, with a confident diagnosis in 53.9% and a provisional diagnosis of high confidence in 27.3% of patients [2]. Even though we did not differentiate levels of confidence regarding diagnosis, it is interesting to note that in 51% of patients with representative cryobiopsy, the definitive/highly compatible histopathological diagnoses led to a concordant and specific MDTD-diagnosis, which is comparable to the percentage of confident diagnosis (53.9%) in the study by Hetzel et al. [2].

In our setting, SLB findings resulted in classifiable ILD diagnoses for 64% of patients, which is lower than reported by the literature [3, 7], however, this might be due to the fact that we performed SLB after non-diagnostic cryobiopsy only, whereas numbers in the literature include results from primary SLB. Importantly, in patients who were not classifiable based on cryobiopsy, subsequent SLB provided a specific diagnosis in only 50%, whereas 50% of patients remained non-classifiable. This is in line with the COLDICE prospective study, where in 50% of the patients with unclassifiable ILD after cryobiopsy, SLB did not change the final diagnosis [1]. Thus, in cases of unclassifiable ILD after cryobiopsy and MDTD, the additional information gained by SLB might be limited in a substantial proportion of patients.

After final diagnostic procedures and MDTD, 16% of our patients remained non-classifiable (Fig. 1B), which is consistent with the literature [8].

In 15% of patients with alternative diagnosis CT patterns, cryobiopsy—integrated into MDTD—led to a final diagnosis of IPF. Thus, cryobiopsy obviated SLB and its risks in a considerable proportion of these patients. Of note, in those patients with a typical UIP pattern on CT scan but a history suggestive of a non-IPF diagnosis, cryobiopsy was diagnostic in 92% of patients, suggesting that specifically in those patients cryobiopsy is a reasonable and safe diagnostic approach.

Our final diagnoses were comparable to the distribution of first choice diagnosis in other cryobiopsy-studies, with IPF and HP being the most common diagnoses (Fig. 1B) [2, 6].

Bleeding rates in our analysis were lower, whereas rates of pneumothorax were comparable to earlier studies [2, 9].

As the diagnostic yield of cryobiopsy is influenced by the number of samples taken [10], and due to the fact that in 12% of our patients samples were not representative, we will increase the number of samples to four per patient in the future.

As in previous studies [2, 6], the major limitation of our analysis is the lack of specific, pre-defined criteria to undergo cryobiopsy. On the other hand, our study represents the real-world practice where decisions for diagnostic procedures are taken stepwise and individually for every patient.

In summary, we demonstrate that the diagnostic yield of cryobiopsy is high if combined with BAL-, radiological- and clinical data, and therefore substantiate the crucial role of cryobiopsy as a diagnostic tool in patients with ILD.

## Data Availability

Due to protection of patient data privacy sharing of data on a publicly available repository is not possible, but data are available from the corresponding author upon request.

## Abbreviations

BAL: Broncho-alveolar lavage
CT: Computed tomography
HP: Hypersensitivity pneumonitis
ILD: Interstitial lung diseases
IPF: Idiopathic pulmonary fibrosis
MDTD: Multidisciplinary team discussion
SLB: Surgical lung biopsy
UIP: Usual interstitial pneumonia
